# Effects of Probiotic Supplementation from Dry-Off Through Early Lactation on Serum Protein Indices and Free Amino Acids in Dairy Cows: A Randomized Field Study

**DOI:** 10.3390/ijms27177860

**Published:** 2026-09-02

**Authors:** Jan Marczuk, Karolina Wrześniewska, Piotr Brodzki, Adam Brodzki, Kenan Sezer, Dawid Tobolski

**Affiliations:** 1 Sub-Department of Internal Diseases of Farm Animals and Horses, Department and Clinic of Animal Internal Medicine, Faculty of Veterinary Medicine, University of Life Sciences in Lublin, Głęboka 30, 20-612 Lublin, Poland; jan.marczuk@up.edu.pl (J.M.); karolina.wrzesniewska@up.edu.pl (K.W.); 2 Department and Clinic of Animal Reproduction, Faculty of Veterinary Medicine, University of Life Sciences in Lublin, Głęboka 30, 20-612 Lublin, Poland; piotr.brodzki@up.edu.pl; 3 Department and Clinic of Animal Surgery, Faculty of Veterinary Medicine, University of Life Sciences in Lublin, Głęboka 30, 20-612 Lublin, Poland; adam.brodzki@up.edu.pl; 4 Department of Internal Medicine, Faculty of Veterinary Medicine, Burdur Mehmet Akif Ersoy University, 15000 Burdur, Turkey; ksezer@mehmetakif.edu.tr; 5 Department of Large Animal Diseases and Clinic, Institute of Veterinary Medicine, Warsaw University of Life Sciences (SGGW), Nowoursynowska 159c, 02-787 Warsaw, Poland

**Keywords:** dairy cow, probiotic, mastitis, amino acids, periparturient period, immune response

## Abstract

The transition from late gestation to lactation is accompanied by marked changes in protein and amino acid metabolism in dairy cows. This randomized field study evaluated whether oral multi-strain probiotic supplementation from dry-off to 12 weeks postpartum affected serum protein indices and circulating free amino acids. Twenty multiparous cows were allocated 1:1 to a control group (CON) or a probiotic-supplemented group (PRO). The longitudinal hematology and serum biochemistry subcohort comprised five cows per group, whereas free amino acids were measured at 7–8 weeks postpartum in 9 CON and 10 PRO cows. At 3–4 weeks postpartum, PRO cows had higher total protein (83.86 vs. 73.98 g/L; *p* = 0.009) and globulin (40.20 vs. 32.80 g/L; *p* = 0.024). At 7–8 weeks postpartum, PRO cows had lower asparagine (13.30 vs. 20.11 μmol/L; *p* = 0.004) and glutamine (280.90 vs. 363.44 μmol/L; *p* = 0.019), and higher aspartic acid (24.80 vs. 6.56 μmol/L; *p* = 0.008) and tryptophan (26.60 vs. 16.67 μmol/L; *p* = 0.045). Probiotic supplementation was associated with differences in selected serum protein indices and amino acids, although the underlying mechanisms remain to be established.

## 1. Introduction

The periparturient period in dairy cows, which encompasses late gestation and early lactation, is a phase of significant metabolic and physiological change initiated by calving and lactogenesis [1,2]. A primary characteristic of this transition is the development of a negative energy balance (NEB), a condition where the high energy demands for milk synthesis exceed the cow’s energy intake from feed [2,3]. To compensate for the energy deficit, the cow mobilizes endogenous reserves, including adipose tissue and skeletal muscle protein. The catabolism creates significant metabolic stress, which increases the animal’s susceptibility to various periparturient diseases, from metabolic disorders like ketosis to infectious conditions such as metritis and mastitis [4,5]. Mastitis, an inflammation of the mammary gland, is a prevalent and economically costly disease in the dairy industry. The condition is caused by pathogenic microorganisms, and its incidence is increased by factors associated with the transition period, including lactation stress, oxidative stress, and periparturient immunosuppression [6,7]. The greatest vulnerability to new intramammary infections occurs at the start of the dry period and immediately before parturition, when the cow’s immune systems are naturally suppressed [8].

The conventional view held that the primary route of mammary gland infection was exogenous, through the invasion of pathogens via the teat canal [9]. While the exogenous route is significant, research also indicates the potential importance of endogenous pathways, particularly the “gut–mammary axis” [10,11]. This concept proposes that disturbances in the gastrointestinal microbiota may impair intestinal epithelial barrier function, increase the passage of microbial components into the circulation, and thereby influence inflammatory responses at distant sites, including the mammary gland [12,13].

The structural and functional integrity of the intestinal barrier depends partly on nutrient availability. Enterocytes use glutamine, glutamate, and asparagine as important metabolic substrates [14,15,16,17,18,19], and amino acids participate in tight-junction maintenance, cell proliferation, and mucosal immune functions [20]. During the transition period, nutrient partitioning toward the mammary gland may alter the availability of amino acids to other tissues [21,22]. Accordingly, circulating amino acid profiles provide an integrated measure of the metabolic adaptations that accompany the transition from gestation to lactation.

Amino acids also support immune-cell metabolism during the periparturient period [20,23,24]. Tryptophan participates in serotonin synthesis and the kynurenine pathway [20], whereas inflammation can alter circulating amino acid concentrations through changes in acute-phase protein synthesis and cellular utilization [25]. Metabolomic studies have identified amino acid patterns associated with subsequent subclinical mastitis [7,26]. These observations provide a rationale for examining whether nutritional interventions during the transition period are accompanied by differences in circulating free amino acids.

Direct-fed microbials (DFMs) have been investigated as non-antibiotic nutritional interventions in ruminants [11,27,28]. Reported effects depend on microbial strain, viability, dose, diet, and herd management [29,30,31,32,33]. Preparations containing lactic acid bacteria and yeast have been associated with changes in rumen fermentation, nutrient use, health, and productivity [29,30,31,32,33,34,35]. The present study evaluated the administered commercial multistrain formulation as a complete consortium under field conditions, providing a pragmatic assessment of its association with host metabolic outcomes.

Limited research has evaluated associations between oral probiotic supplementation and host protein and amino acid profiles during the transition from late gestation to lactation. We hypothesized that supplementation with a multistrain probiotic containing lactic acid bacteria and *Saccharomyces cerevisiae*, administered from the dry period through the first three months of lactation, would be associated with changes in systemic protein metabolism. The primary objective was to evaluate serum total protein, albumin, calculated globulins, and the complete measured panel of free amino acids. Udder-health and milk-production variables were included to provide clinically relevant context for the metabolic findings. It remains unclear how probiotic supplementation affects the systemic amino acid profile during the transition period because most available studies have focused on productivity and rumen-related outcomes. Accordingly, the present randomized single-herd pilot study was designed to identify potential treatment-associated metabolic patterns and to generate more specific hypotheses about probiotic mechanisms for future studies.

## 2. Results

### 2.1. Hematological Profile

The hematological parameters for both the probiotic-supplemented (PRO) and control (CON) groups throughout the study period are summarized in Supplementary Table S4. The linear mixed-effects model revealed a significant main effect of probiotic treatment on red blood cell count (RBC), hemoglobin (HGB), hematocrit (HCT), white blood cell count (WBC), band and segmented neutrophils, lymphocytes, mean corpuscular hemoglobin concentration (MCHC), and mean corpuscular volume (MCV) (*p* < 0.05). A significant main effect of time was observed for HCT, HGB, WBC, and segmented neutrophils (*p* < 0.05). A significant group-by-time interaction was detected for HGB, lymphocytes, mean corpuscular hemoglobin (MCH), and MCHC (*p* < 0.05).

Post hoc comparisons of erythrocyte parameters showed higher HGB concentrations in the PRO group at 10–14 days prepartum (6.51 vs. 6.05 mmol/L; *p* = 0.036) and at 3–4 weeks postpartum (6.14 vs. 5.16 mmol/L; *p* = 0.005). Trends toward higher HGB (*p* = 0.059), RBC count (*p* = 0.070), and HCT (*p* = 0.081) were observed at 5–10 days postpartum, and a trend toward higher HCT was observed at 3–4 weeks postpartum (*p* = 0.066).

A trend toward higher MCHC was observed in the PRO group at 3–4 weeks postpartum (*p* = 0.053), whereas a trend toward lower MCHC was observed at 11–12 weeks postpartum (*p* = 0.069). No between-group differences were observed for MCV or platelet count at an individual time point.

Regarding the leukocyte profile, WBC count was higher in the PRO group at 5–10 days postpartum (8.70 vs. 5.78 × 10^9^/L; *p* = 0.008). Segmented neutrophil counts were higher at 10–14 days prepartum (4.14 vs. 2.13 × 10^9^/L; *p* = 0.012) and 5–10 days postpartum (4.26 vs. 2.07 × 10^9^/L; *p* = 0.039), and band neutrophils showed a trend toward a higher count at 5–10 days postpartum (*p* = 0.075). The lymphocyte count was lower in the PRO group at 10–14 days prepartum (3.70 vs. 5.40 × 10^9^/L; *p* = 0.041). No between-group differences were observed for eosinophils.

### 2.2. Serum Protein Metabolism

**Figure 1:**
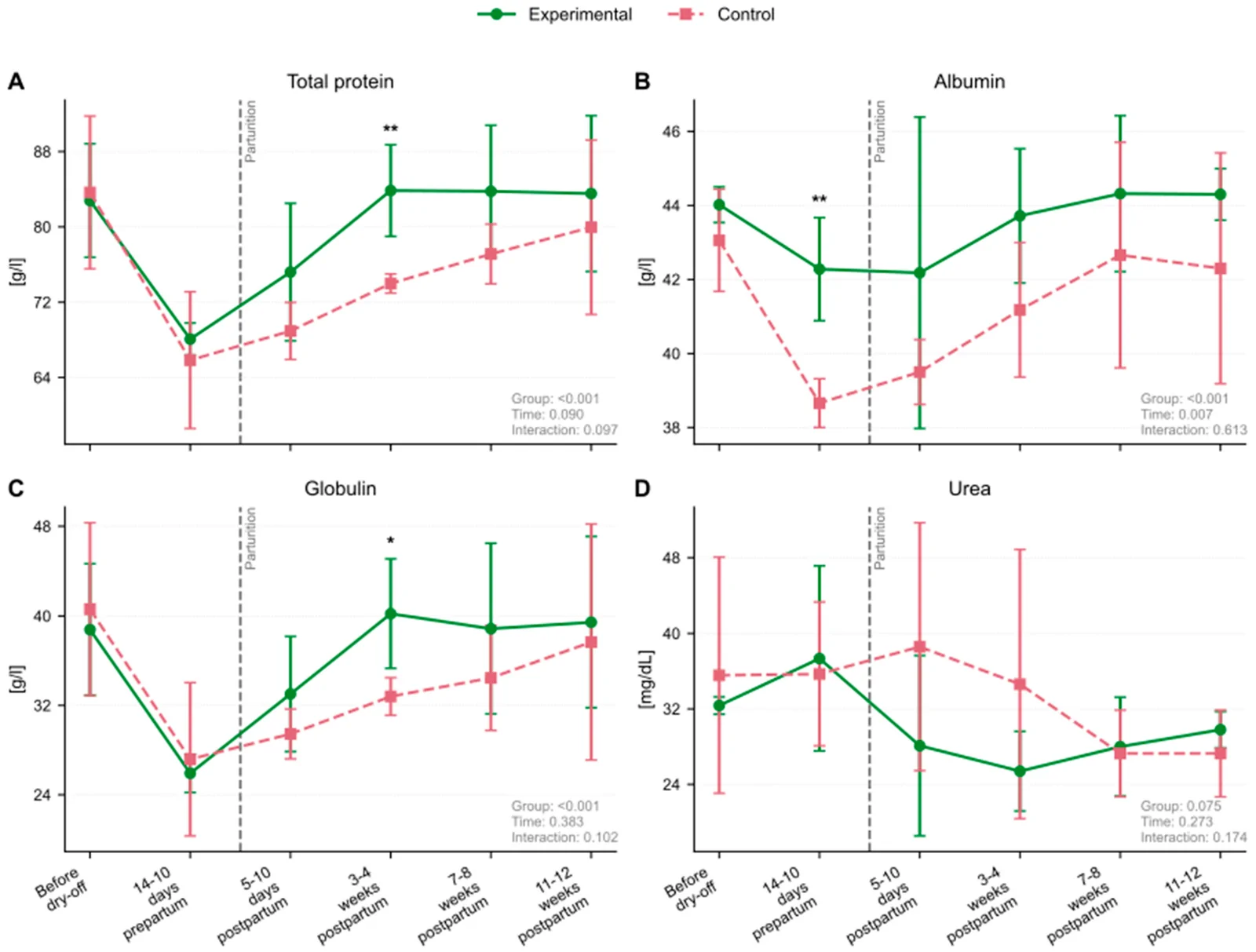
Longitudinal changes in serum protein metabolites in response to probiotic supplementation. Line graphs show serum concentrations (mean ± SD) for the probiotic-supplemented (PRO) and control (CON) groups at six time points relative to calving. Panels display: (**A**) total protein [g/L], (**B**) albumin [g/L], (**C**) globulin [g/L], and (**D**) urea [mg/dL]. The vertical dashed line indicates parturition. Asterisks (* *p* < 0.05, ** *p* < 0.01) denote between-group differences at a specific time point. The *p* values for the main effects of group and time and the group-by-time interaction are shown on each panel.

The results for serum protein metabolites are presented in Supplementary Table S5. The analysis indicated a significant main effect of treatment group on total protein, albumin, and globulin concentrations (*p* < 0.001). A significant main effect of time was found for albumin (*p* = 0.007) and the albumin-to-globulin ratio (*p* < 0.001), with a trend for total protein (*p* = 0.090). No significant group × time interactions were detected; trends were observed for total protein (*p* = 0.097) and globulin (*p* = 0.102) (Figure 1).

At 3–4 weeks postpartum, PRO cows had higher total protein (83.86 vs. 73.98 g/L; *p* = 0.009) and calculated globulin concentrations (40.20 vs. 32.80 g/L; *p* = 0.024) than CON cows. The corresponding time-specific Hedges’ g values (PRO minus CON) were 2.54 (95% CI, 0.85 to 4.16) for total protein and 1.83 (95% CI, 0.37 to 3.22) for calculated globulin (Supplementary Table S9).

Albumin concentration was higher in the PRO group at 10–14 days prepartum (42.28 vs. 38.66 g/L; *p* = 0.008), with a trend at 3–4 weeks postpartum (*p* = 0.058). No between-group differences were observed for urea at an individual time point.

### 2.3. Serum Free Amino Acid Profile

**Figure 2:**
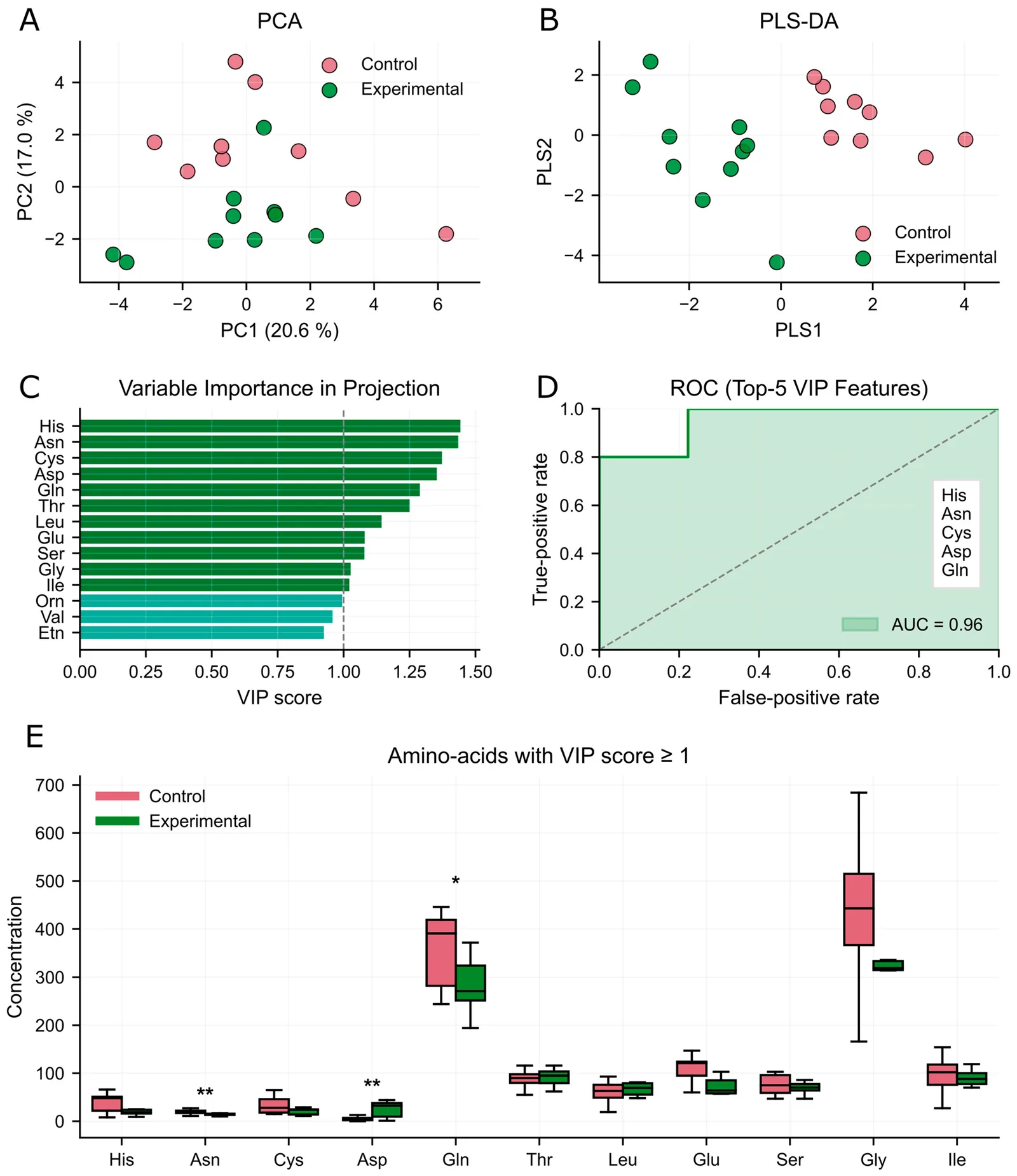
Multivariate and univariate analyses of serum free amino acids at 7–8 weeks postpartum in control (CON; *n* = 9) and probiotic-supplemented (PRO; *n* = 10) cows. (**A**) PCA scores plot. (**B**) PLS-DA scores plot. (**C**) VIP scores. (**D**) Receiver operating characteristic curve based on the five highest-ranked VIP variables. (**E**) Boxplots of amino acids with VIP scores ≥1.0. Asterisks denote between-group *p* values (* *p* < 0.05; ** *p* < 0.01).

Serum free amino acid profiles were analyzed at 7–8 weeks postpartum to characterize established lactation. Principal component analysis (PCA) showed that the first two principal components explained 20.6% and 17.0% of the total variance, respectively, with partial separation between the PRO and CON groups (Figure 2A). Supervised partial least squares-discriminant analysis (PLS-DA) showed distinct clustering and separation between groups in the analyzed dataset (Figure 2B).

Variable importance in projection (VIP) scores ≥1.0 were observed for histidine, asparagine, cysteine, aspartic acid, glutamine, threonine, leucine, glutamic acid, serine, glycine, and isoleucine (Figure 2C). A model based on the five highest-ranked variables (histidine, asparagine, cysteine, aspartic acid, and glutamine) yielded an area under the receiver operating characteristic curve of 0.96 (Figure 2D,E).

Univariate analysis showed a higher tryptophan concentration in the PRO group (26.60 vs. 16.67 μmol/L; *p* = 0.045; Benjamini–Hochberg *q* = 0.218). No other essential amino acid differed between groups, and the sum of essential amino acids was similar (*p* = 0.903). The standardized difference for tryptophan was Hedges’ *g* = 0.73 (95% CI, −0.17 to 1.61; PRO minus CON; Supplementary Table S10).

Among nonessential amino acids, aspartic acid was higher in the PRO group (24.80 vs. 6.56 μmol/L; *p* = 0.008; *q* = 0.092), whereas asparagine (13.30 vs. 20.11 μmol/L; *p* = 0.004; *q* = 0.092) and glutamine (280.90 vs. 363.44 μmol/L; *p* = 0.019; *q* = 0.146) were lower. Glutamic acid and ornithine each had a *p* value of 0.057 (*q* = 0.218) (Supplementary Table S6). The corresponding Hedges’ g values were 1.36 (95% CI, 0.37 to 2.32) for aspartic acid, −1.52 (95% CI, −2.50 to −0.50) for asparagine, and −1.18 (95% CI, −2.11 to −0.22) for glutamine (Supplementary Tables S6 and S10).

### 2.4. Milk Production and Udder Health

Milk production and composition during the first 105 days in milk (DIM) are presented in Supplementary Table S7 and S8. In the longitudinal subcohort (CON, *n* = 5; PRO, *n* = 5), mean cumulative milk yield and cumulative fat yield per cow were numerically lower in the PRO group, but the between-group differences were not statistically significant. Mean cumulative milk protein yield per cow was lower in the PRO group (102.60 ± 11.06 kg/cow) than in the CON group (121.20 ± 9.38 kg/cow; *p* < 0.05). Mean milk fat, protein, and dry matter contents did not differ between groups.

Monthly test-day somatic cell count (SCC) records showed that four of five CON cows and two of five PRO cows had at least one SCC value >300,000 cells/mL during the first two months postpartum (two-sided Fisher’s exact test, *p* = 0.524). These observations are reported as SCC-defined elevations. Milk production, composition, and SCC were monitored during the broader first-105-DIM observation period.

## 3. Discussion

This randomized single-herd pilot field study was designed to detect large metabolic effects and exploratory patterns in serum protein indices and circulating free amino acids. Its findings should therefore be interpreted with caution and as hypothesis-generating. Within these limits, probiotic supplementation was associated with higher total ds and calculated globulin concentrations at 3–4 weeks postpartum and with an exploratory amino acid profile at 7–8 weeks postpartum. A lower frequency of SCC-defined elevations was observed in the PRO group during the first two months postpartum, but this comparison was not statistically significant. The observed patterns may assist the design and power calculations of larger validation studies.

The increase in total protein and calculated globulin concentrations represents a treatment-associated pattern in circulating protein status during early lactation. Because globulins include immunoglobulins and other proteins involved in inflammatory and transport processes, this finding provides a biologically plausible hypothesis for follow-up rather than evidence that immune modulation occurred. The higher leukocyte and neutrophil counts observed around parturition likewise warrant direct mechanistic evaluation. The numerical SCC pattern is not interpreted as evidence of a health benefit [29,32,36,37].

The nominal differences in circulating asparagine, glutamine, and aspartic acid should be interpreted as exploratory because none of the individual amino acids met the false-discovery-rate-adjusted threshold. Their directions and standardized effect estimates nevertheless identify candidates for mechanistic follow-up concerning enterocyte metabolism, nitrogen transfer, and immune-cell function [20]. The present findings are consistent with prior observations that circulating amino acid profiles vary with metabolic and mammary-health status [7,25,26], but they do not establish altered tissue utilization, interorgan exchange, or catabolism.

Tryptophan was nominally higher in the PRO group (q = 0.218; Hedges’ g = 0.73, 95% CI, −0.17 to 1.61) and contributed to the multivariate separation between groups. Because tryptophan participates in serotonin synthesis and the kynurenine pathway [20], this pattern provides a rationale for direct measurement of downstream metabolites in future studies. It does not demonstrate altered serotonin or kynurenine-pathway activity, immune modulation, or a favorable health effect. Previous associations between lower tryptophan concentrations and subsequent subclinical mastitis [7,22,26] should therefore be regarded as biological context rather than confirmation of a mechanism in the present study.

Production responses were variable. Total milk and fat yields were numerically lower in the PRO group, and cumulative milk protein yield was lower, whereas mean milk protein content was similar between groups. Thus, the metabolic differences did not translate into a clear increase in production during the monitored period. This divergence emphasizes that circulating metabolic indices and productive performance represent related but distinct outcome domains. Previous studies have also reported variable production responses to probiotics [31,35,38], likely reflecting differences in formulation, dose, diet, stage of lactation, and herd management.

Consistent with its pilot design, the study should be interpreted in view of its field setting and outcome-specific sample sizes. The a priori calculation addressed the primary amino acid domain and targeted a large, standardized difference (Cohen’s *d* = 1.33), whereas the nested longitudinal subcohort included five cows per group. This design was appropriate for detecting large metabolic signals and characterizing repeated within-cow trajectories, but it provided limited precision for modest production and udder health effects. Retaining the reported raw *p* values preserves analytical transparency and allows readers to apply Bonferroni, Holm, false-discovery-rate, or another correction according to their selected definition of the comparison family. These results should therefore be considered together with the global linear mixed-effects model tests and the consistency of temporal response patterns. In contrast, the 23 individual amino acids constituted a clearly defined analytical family; consequently, Benjamini–Hochberg-adjusted *q* values were calculated and reported alongside the raw *p* values.

The study was conducted in one herd, which limits generalizability. During the dry period, the supplement was delivered under group-feeding conditions; the assigned dose was calculated for the treatment pen and mixed using a mineral carrier, but individual consumption was not measured. Individual dry matter and water intake were also not quantified. The complete commercial consortium was evaluated at one fixed dose, so component-specific and dose–response effects cannot be separated. Prela HP was administered identically to both groups, although a probiotic-by-supplement interaction was not tested. Amino acids were measured once at 7–8 weeks postpartum and therefore cannot be linked temporally to the protein differences observed at 3–4 weeks. The multivariate model was evaluated in the derivation dataset without external validation. SCC was assessed from monthly test-day records; persistence was not required, bacteriological testing was not performed, and age and parity were not included as covariates. The exact number of available SCC samples per cow could not be reconstructed from the records available for this revision. Circulating leukocyte counts may also reflect parturition-associated physiological changes, metabolic stress, or inflammatory recruitment rather than treatment-specific immune competence. Finally, intestinal permeability, rumen or gut microbiota, microbial protein synthesis, inflammatory and oxidative-stress markers, kynurenine-pathway metabolites, immunoglobulin classes, and leukocyte function were not measured. These aspects should be addressed in larger multi-herd studies with individual intake monitoring and direct mechanistic endpoints.

## 4. Materials and Methods

### 4.1. Ethical Approval

All procedures involving animals were conducted in accordance with European legislation, namely Directive 2010/63/EU of the European Parliament and of the Council of 22 September 2010 on the protection of animals used for scientific purposes. The study protocol was approved by the Local Ethics Committee for Animal Experiments at the University of Life Sciences in Lublin (No. 41/2014). The farm owner provided informed consent prior to the commencement of the study.

### 4.2. Animal Management

The study was conducted during the summer–autumn period of 2014 on a commercial dairy farm located in Krasienin, Lublin Voivodeship, Poland. The source herd comprised 62 lactating cows, which were a genetic mix of Polish Black-and-White and Holstein-Friesian breeds. The cows were between 3 and 6 years of age, with an average body weight of 500–600 kg. Based on routine herd records, the 305-day lactation yield ranged from 6483 to 10,443 kg of milk, with average milk fat and protein contents of 3.62–4.13% and 3.38–3.50%, respectively.

The farm utilized a dual housing system according to production stage. During the dry period, non-lactating cows were housed in a free-stall system, allowing free movement and expression of natural behaviors. After parturition, lactating cows were transferred to a tie-stall barn, enabling individual monitoring of health status, feed intake, and experimental treatments. Lactating cows were milked twice daily at 12-h intervals using a pipeline milking system. Water was available ad libitum throughout the study period.

The nutritional program for lactating cows was based on a corn silage-based total mixed ration (TMR) formulated to meet the nutritional requirements of a cow producing 20 kg of milk per day. The basal TMR consisted of maize silage, haylage, oat straw, corn grain silage, cereal meal (triticale, oats, barley), soybean meal, rapeseed meal, a commercial protein concentrate (Protamilk; Sano, Sękowo, Poland), wet sugar beet pulp, brewers’ grains, molasses, and a vitamin–mineral premix containing sodium bicarbonate and yeast metabolites. The as-fed composition of the TMR per head and the inclusion rate of each ingredient are presented in Supplementary Table S1. For cows exceeding a daily milk yield of 20 kg, an additional 1 kg of concentrate was provided for each 2 kg of milk produced above this threshold, administered individually after milking.

Dry cows received a ration based on the same forages, adjusted to their physiological stage, and were additionally supplemented with a bitter-salts dry cow product (Prela HP; Sano, Sękowo, Poland) at 2 kg/head/day. This supplement was offered from approximately 6–7 weeks before the expected calving date until calving. The chemical composition of Prela HP, including crude protein (with non-protein nitrogen), crude ash, crude fiber, sugar, starch, crude fat, and macroelements (Ca, P, Na, Mg, S), is summarized in Supplementary Table S2. Prela HP was administered identically to PRO and CON cows; because no factorial treatment arm was included, a probiotic-by-supplement interaction could not be estimated.

### 4.3. Experimental Groups and Probiotic Intervention

The randomized, controlled field study enrolled 20 clinically healthy, multiparous dairy cows from a single commercial herd. To reduce baseline imbalances, cows were screened for eligibility and matched before allocation based on parity (2–5 lactations), previous lactation milk yield, and body condition score (BCS). Within each matched pair, one cow was randomly assigned to the probiotic-supplemented group (PRO) and the other to the control group (CON) using a simple random number generator, with a 1:1 allocation ratio (Supplementary Table S3). The target sample size at enrollment was 10 cows per group. Housing, feeding, and milking routines were identical across groups within each production stage; the only planned treatment difference was administration of the probiotic to cows in the PRO group. Treatment delivery was not blinded under field conditions.

**Figure 3:**
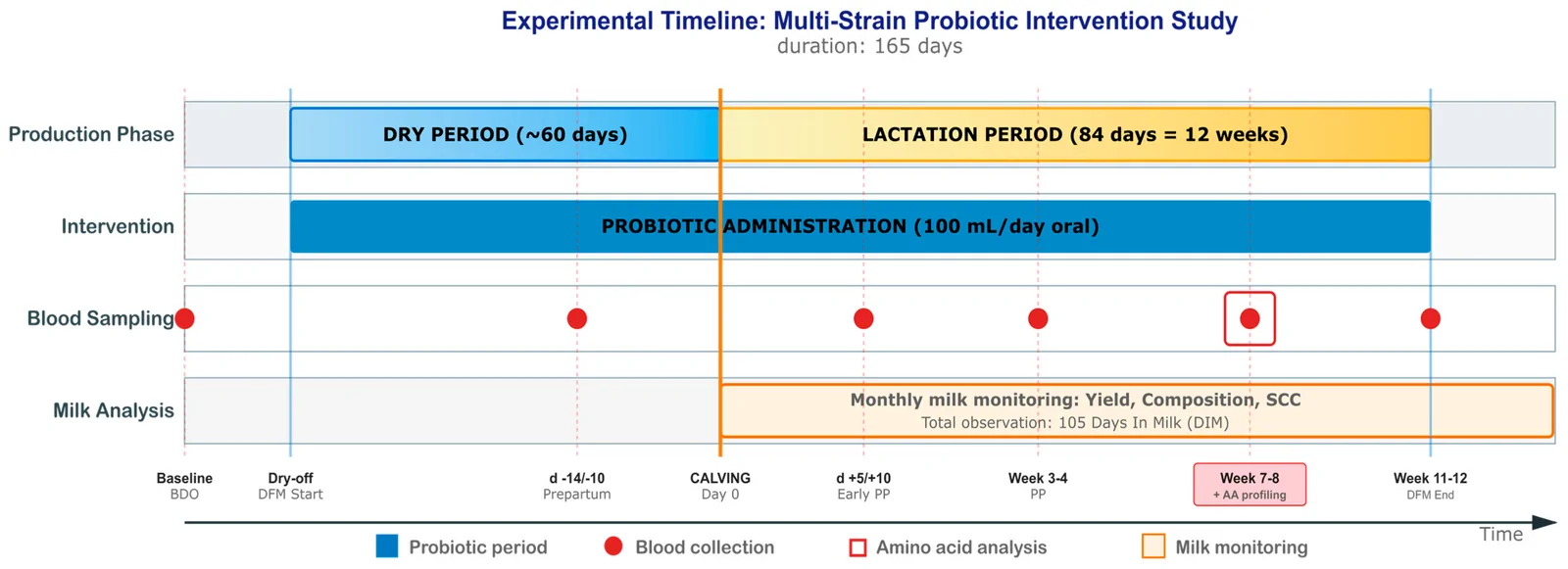
Experimental timeline. Probiotic supplementation began at dry-off, approximately 60 days before expected calving, and continued through 12 weeks (84 days) postpartum. Blood for longitudinal hematology and serum biochemistry was collected from CON (*n* = 5) and PRO (*n* = 5) cows before dry-off, at 10–14 days prepartum, and at 5–10 days, 3–4 weeks, 7–8 weeks, and 11–12 weeks postpartum. Free amino acids were measured once at 7–8 weeks postpartum in CON (*n* = 9) and PRO (*n* = 10) cows. Milk yield, milk composition, and SCC were monitored for the first 105 DIM in the longitudinal sub-cohort. The longitudinal subcohort was nested within the randomized cohort, and each outcome was analyzed within its stated analytic set.

To accommodate different sampling intensities while preserving the randomized study structure, two prespecified analytic cohorts were defined. The sample size calculation focused on serum free amino acid concentrations as the primary metabolic endpoints. Based on variability reported in comparable studies on bovine nitrogen metabolism [39], a group size of 10 cows was estimated to provide ≥80% power to detect a standardized effect size (Cohen’s d) of 1.33 at a two-sided significance level (α = 0.05). The randomized cohort of 20 cows (CON, *n* = 10; PRO, *n* = 10) served as the basis for all analyses of free serum amino acid concentrations. A nested longitudinal subcohort was selected for repeated phenotyping over time. From each treatment group, 5 cows were randomly chosen, yielding a repeated measures subcohort of 10 cows (CON, *n* = 5; PRO, *n* = 5). For cows included in the longitudinal subcohort, production traits, hematological indices, primary biochemical parameters, and mammary health indicators were recorded at every scheduled sampling time point. A single blood sample for determination of free serum amino acid concentrations was collected from each cow once between the seventh and eighth week postpartum, so that amino acid profiling retained the full randomized sample size targeted by the initial power analysis. During follow-up, one cow in the CON group was culled for reasons unrelated to the dietary intervention; the final amino acid dataset therefore comprised samples from 9 cows in the CON group and 10 cows in the PRO group, whereas repeated-measures analyses were restricted to the longitudinal subcohort with complete series of measurements (Figure 3).

The longitudinal subcohort was nested within the full randomized cohort and did not constitute an independent set of animals. Analyses were performed within their stated outcome domains; no correlations were calculated between amino acid concentrations and longitudinal serum proteins, SCC, or production variables.

The probiotic intervention was standardized across PRO cows, with administration procedures adapted to housing conditions. Beginning at dry-off (approximately 60 days before the expected calving date) and continuing through 12 weeks postpartum, each PRO cow was assigned an intended once-daily 100 mL dose of a multistrain liquid probiotic (EM Probiotic, Greenland Technologia EM^®^, Janowiec, Poland). During the dry period, cows were maintained in free-stall housing in separate treatment pens. The probiotic was premixed into a mineral carrier and incorporated into the TMR; the total volume added was calculated by multiplying 100 mL by the number of cows in the PRO pen. Individual feed intake was not quantified during group feeding. During lactation in tie-stall housing, the probiotic was top-dressed and manually mixed into each PRO cow’s individual morning feed allotment. The formulation contained *Lactobacillus plantarum* and *Lactobacillus casei*, each at 5 × 10^6^ CFU/mL, and *Saccharomyces cerevisiae* at 3 × 10^3^ CFU/mL. Control cows received 100 mL of potable water by the corresponding route. The study evaluated the complete commercial formulation at one fixed assigned dose.

### 4.4. Blood Sample Collection and Processing

Venous blood samples were collected in the morning, prior to feed delivery, via jugular venipuncture using standardized, low-stress handling protocols. Blood collection was scheduled at six physiological time points: baseline before dry-off, 10–14 days before expected calving, 5–10 days postpartum, 3–4 weeks postpartum, 7–8 weeks postpartum, and 11–12 weeks postpartum. For free amino acid analysis, a single serum sample was collected at 7–8 weeks postpartum from each cow in the full randomized cohort to characterize established lactation. Blood was collected into K2-EDTA tubes for hematological analyses and into serum-separator tubes with a clot activator for biochemical determinations (BD Vacutainer^®^, Becton, Dickinson and Company, Franklin Lakes, NJ, USA). EDTA-anticoagulated tubes were immediately placed on ice and analyzed within four hours. Serum tubes were allowed to clot at ambient temperature for 30 min and centrifuged at 1500× *g* for 15 min at 4 °C (MPW M. Diagnostic centrifuge, MPW MED. Instruments, Warsaw, Poland). Serum was aliquoted and stored at −80 °C pending analysis (Figure 3).

### 4.5. Hematological Analysis Procedures

Hematological parameters were determined in whole blood using the scil Vet abc Plus automated hematology analyzer (scil animal care company GmbH, Viernheim, Germany). The analyzer used impedance-based cell counting for erythrocytes, leukocytes, and platelets and photometric hemoglobin measurement. The analytical panel included white blood cell count (WBC, ×10^9^/L), red blood cell count (RBC, ×10^12^/L), hemoglobin (HGB, mmol/L), hematocrit (HCT, %), mean corpuscular volume (MCV, fL), mean corpuscular hemoglobin (MCH, pg), mean corpuscular hemoglobin concentration (MCHC, mmol/L), and platelet count (PLT, ×10^9^/L). Peripheral blood smears were air-dried, stained with May–Grünwald–Giemsa stain (Merck KGaA, Darmstadt, Germany), and examined under oil immersion at 1000× magnification. At least 100 leukocytes per smear were classified morphologically as band neutrophils, segmented neutrophils, eosinophils, basophils, monocytes, or lymphocytes, and absolute counts were calculated. Lot-specific calibration and quality-control identifiers were unavailable for retrospective reporting.

### 4.6. Serum Protein Metabolism Analysis

After completion of sampling, stored serum aliquots were thawed once and analyzed in one batch to minimize inter-assay variation. Total protein, albumin, and urea were measured using a Pentra 400 automated biochemical analyzer with manufacturer-supplied reagent systems (Horiba ABX, Warsaw, Poland). Total protein was measured by the biuret colorimetric method, albumin by the bromocresol-green colorimetric method, and urea by the urease–glutamate dehydrogenase enzymatic ultraviolet method. Lot-specific reagent, calibrator, and control identifiers were unavailable for retrospective reporting. Globulin was calculated as total protein minus albumin, and the albumin-to-globulin ratio was calculated from the resulting concentrations (Figure 1).

### 4.7. Free Amino Acid Determination

Serum free amino acid concentrations were measured in samples collected at 7–8 weeks postpartum and analyzed in one continuous batch. Individual amino acids were quantified by ion-exchange chromatography using an Ingos AAA-400 automatic amino acid analyzer (Ingos s.r.o., Prague, Czech Republic). Proteins were precipitated by mixing 1 mL of serum with 1 mL of 6.0% buffered sulfosalicylic acid prepared in lithium citrate buffer (pH 2.9). After centrifugation at 12,000 rpm for 15 min in an MPW 250 centrifuge (MPW MED. Instruments, Warsaw, Poland), the protein-free supernatant was separated on an Ostion LG FA analytical column (Tessek s.r.o., Prague, Czech Republic; 3 mm × 200 mm). Five 0.2 M lithium citrate buffers were used for gradient elution (pH 2.9, 3.1, 3.35, 4.05, and 4.9). The column temperature was maintained at 38–39 °C for acidic and basic amino acids and at 59–60 °C for neutral amino acids. Post-column derivatization used 0.2% ninhydrin and 0.05% stannous chloride in 0.2 M acetate buffer (pH 5.5), with photometric detection at 440 and 570 nm. Amino acids were identified by comparison of retention times with authenticated external physiological amino acid standards supplied by Ingos s.r.o.; peak integration and quantification used the integrated detector and mikro software, version 1.8.0 (Ingos s.r.o.). The manufacturer-reported instrument sensitivity was <50 pmol at a signal-to-noise ratio of 5. The analytical panel comprised nine essential amino acids (histidine, isoleucine, leucine, lysine, methionine, phenylalanine, threonine, tryptophan, and valine), ten nonessential amino acids (alanine, arginine, asparagine, aspartic acid, cysteine, glutamine, glutamic acid, glycine, serine, and tyrosine), and four related metabolites (citrulline, ethanolamine, ornithine, and taurine).

### 4.8. Milk Production Monitoring

Individual milk production and composition were assessed at monthly test days under the official AT4 recording protocol during the first 105 DIM in collaboration with the Polish Federation of Cattle Breeders and Dairy Farmers (Warsaw, Poland). Recorded variables were cumulative and mean daily milk yield, fat yield and content, protein yield and content, milk dry matter yield and content, and SCC. For the cow-level SCC comparison during the first two months postpartum, a cow was classified as having an SCC-defined elevation if at least one available monthly test-day sample exceeded 300,000 cells/mL. Persistence across consecutive samples was not required, and bacteriological testing was not performed. The SCC monitoring window extended to 105 DIM, whereas the reported 4/5 CON versus 2/5 PRO comparison pertains to the first two postpartum months.

### 4.9. Statistical Analysis

Statistical analyses were performed in Python, version 3.9, using pandas (version 3.0.5) for data management, statsmodels (version 0.15.0) for statistical modeling, SciPy (version 1.18.1) for statistical tests, scikit-learn (version 1.9.0) for multivariate analysis, and matplotlib (version 3.11.1) for visualization. Raw *p* values <0.05 were used for the original comparisons. For the 23 individual amino acid comparisons, Benjamini–Hochberg false-discovery-rate-adjusted *q* values were additionally calculated. Data are presented as mean ± standard deviation.

For longitudinal hematological and serum protein data, linear mixed-effects models included treatment group, time, and the group × time interaction as fixed effects and cow as a random intercept. Likelihood-ratio tests were used for global fixed effects. Time-specific post hoc comparisons between groups used Welch’s *t* test or the Mann–Whitney *U* test according to the Shapiro–Wilk assessment of normality; paired *t* tests or Wilcoxon signed-rank tests were used for within-group temporal comparisons when applicable. No multiplicity correction was applied to the time-specific post hoc comparisons. The reported raw *p* values are retained so that readers can apply an adjustment according to their selected definition of the comparison family.

For amino acid profiles, scaled data were explored using principal component analysis and supervised PLS-DA. VIP scores ≥1.0 identified variables contributing to separation. Receiver operating characteristic performance was estimated for the five highest-ranked variables. Individual amino acids were compared between groups using Welch’s *t* test or the Mann–Whitney *U* test according to distributional assessment. Benjamini–Hochberg correction was applied across the 23 individual amino acids, and both raw *p* values and adjusted *q* values are reported. The difference in the number of cows with an SCC-defined elevation was evaluated using a two-sided Fisher’s exact test.

To quantify the direction and magnitude of between-group differences, Cohen’s d was calculated from the pooled standard deviation for all time-specific serum protein indices, individual amino acids and amino acid summary variables, and milk production and composition outcomes. Hedges’ g was then obtained using the small-sample correction J = 1 − 3/[4(nPRO + nCON) − 9], with positive values indicating higher values in PRO and negative values indicating lower values in PRO. Ninety-five percent confidence intervals for Hedges’ g were obtained by inverting the noncentral t distribution and applying the same correction. These summary-statistic-based estimates are reported separately in Supplementary Tables S9–S11. For longitudinal outcomes, they represent descriptive time-specific standardized contrasts and do not replace the linear mixed-effects models; where rank-based tests were used, the standardized mean differences supplement rather than replace the original inferential test.

## 5. Conclusions

In this randomized single-herd pilot field study, multistrain probiotic supplementation from dry-off through early lactation was associated with differences in serum protein indices and circulating free amino acid profiles. Probiotic-supplemented cows had higher total protein and calculated globulin concentrations at 3–4 weeks postpartum and a distinct pattern of asparagine, glutamine, aspartic acid, and tryptophan concentrations at 7–8 weeks postpartum. A lower frequency of SCC-defined elevations was also observed in probiotic-supplemented cows during the first two months postpartum. These findings support further investigation of probiotic-associated immunometabolic changes under commercial dairy conditions. Nevertheless, the observed patterns (higher globulin, a trend toward tryptophan changes) merit further exploration as hypothesis-generating strategies for future studies with a larger sample.

## Data Availability

The original contributions presented in this study are included in the article/Supplementary Material. Further inquiries can be directed to the corresponding author.

## Supplementary Materials

The following supporting information can be downloaded at: https://www.mdpi.com/article/10.3390/ijms27177860/s1.

## Abbreviations

The following abbreviations are used in this manuscript:

A/G	Albumin-to-globulin ratio
AA	Amino acid
ALB	Albumin
AUC	Area under the curve
BCAA	Branched-chain amino acids
BCS	Body condition score
BDO	Before dry-off
CFU	Colony-forming units
CON	Control group
DFM	Direct-fed microbial
DIM	Days in milk
EAA	Essential amino acids
GLOB	Globulin
HCT	Hematocrit
HGB	Hemoglobin
IDO	Indoleamine 2,3-dioxygenase
LMM	Linear mixed-effects model
MCH	Mean corpuscular hemoglobin
MCHC	Mean corpuscular hemoglobin concentration
MCV	Mean corpuscular volume
NEAA	Non-essential amino acids
NEB	Negative energy balance
PCA	Principal component analysis
PLS-DA	Partial least squares-discriminant analysis
PLT	Platelet count
PRO	Probiotic-supplemented group
RBC	Red blood cell count
ROC	Receiver operating characteristic
SCC	Somatic cell count
SD	Standard deviation
TAA	Total amino acids
TMR	Total mixed ration
TP	Total protein
VIP	Variable importance in projection
WBC	White blood cell count
