# Supplementary material for: Effects of Probiotic Supplementation from Dry-Off Through Early Lactation on Serum Protein Indices and Free Amino Acids in Dairy Cows: A Randomized Field Study
Source: Int J Mol Sci. 2026 Sep 2;27(17):7860. doi: 10.3390/ijms27177860 (PMC13566735; doi:10.3390/ijms27177860)
Supplement: Supplementary file 1 [file ijms-27-07860-s001.zip › ijms-4483176-supplementary.pdf]

Supplementary Table 1. Composition of the total mixed ration (TMR) per head/day.

Ingredient	Quantity (kg/head/day)	Inclusion rate (% as-fed)
Maize silage	22.58	42.41
Sugar beet pulp	9.68	18.18
Haylage	4.58	8.60
Brewers grains	4.84	9.09
Corn grain silage	3.23	6.07
Cereal meal (triticale, oats, barley)	3.23	6.07
Straw	0.97	1.82
Molasses	0.97	1.82
Soybean meal	0.81	1.52
Rapeseed meal	0.81	1.52
Protein concentrate	0.81	1.52
Hay	0.32	0.60
Vitamin–mineral premix	0.19	0.36
Sodium bicarbonate	0.11	0.21
Yeast metabolites	0.11	0.21
Total	53.24 kg/head/day	100%

Supplementary Table 2. Composition of the dry cow supplement (Prela HP; Sano, Sękowo, Poland).

Nutrient	Content (%)
Crude protein (CP)	34.0 (incl. 5.0 NPN)
Crude ash	18.0
Crude fiber	6.5
Sugar	6.0
Starch	5.0
Crude fat	2.5
Calcium (Ca)	3.4
Magnesium (Mg)	1.6
Sodium (Na)	1.0
Phosphorus (P)	0.95
Sulfur (S)	0.9

Supplementary Table 3. Baseline characteristics (mean ± SD) in control (CON) and probiotic-supplemented (PRO) cows at enrollment.

Parameter	PRO (n = 10)	CON (n = 10)	p-value
Parity (lactation no.)	2.8 ± 0.42	3.2 ± 1.23	0.34
Previous 305-d milk yield (kg)	8370.8 ± 1398.3	8021.0 ± 1735.3	0.63
BCS (1–5 scale)	3.73 ± 0.22	3.70 ± 0.20	0.79

Supplementary Table 4. Hematological parameters (mean ± SD) in control (CON; *n* = 5) and probiotic-supplemented (PRO; *n* = 5) cows at different time points relative to calving.

	Group	BDO	d-14	d+7	w+4	w+8	w+12
RBC [10^{12}/l]	CON	7.06 ± 0.95	6.40 ± 0.28	5.58 ± 0.62	5.57 ± 0.63	6.29 ± 0.83	6.71 ± 0.97
	PRO	7.44 ± 0.46	6.71 ± 0.70	6.28 ± 0.38	6.49 ± 1.26	6.46 ± 0.61	7.01 ± 0.56
HCT [%]	CON	33.28 ± 4.23	29.20 ± 2.08	25.48 ± 3.28	25.14 ± 2.91	28.40 ± 3.15	28.80 ± 3.43
	PRO	34.96 ± 2.95	31.04 ± 1.83	29.14 ± 2.36	28.44 ± 1.57	29.20 ± 1.73	31.22 ± 1.75
HGB [mmol/l]	CON	7.28 ± 0,83	6.05 ± 0,28	5.39 ± 0,60	5.16 ± 0,44	6.25 ± 0,62	6.36 ± 0,67
	PRO	7.08 ± 0,53	6.51 ± 0,29	6.16 ± 0.48	6.14 ± 0.35	6.34 ± 0.32	6,71 ± 0.35
MCHC [mmol/l]	CON	21.91 ± 0.84	20.79 ± 0.57	21.20 ± 0.94	20.58 ± 0.84	21.88 ± 0.40	21.88 ± 0.40
	PRO	20.23 ± 0.36	20.96 ± 0.34	21.20 ± 0.76	21.60 ± 0.21	21.71 ± 0.63	21.27 ± 0.49
MCV [fl]	CON	47.20 ± 1.48	45.40 ± 1.95	45.80 ± 1.64	45.00 ± 1.87	45.20 ± 2.68	45.20 ± 2.68
	PRO	47.00 ± 3.46	47.00 ± 2.92	46.40 ± 3.29	44.80 ± 6.61	45.40 ± 3.78	45.20 ± 1.92
MCH [pg]	CON	16.70 ± 0.87	14.82 ± 1.16	15.60 ± 1.07	14.96 ± 0.67	15.96 ± 1.03	15.96 ± 1.03
	PRO	15.34 ± 0.99	15.58 ± 1.07	15.84 ± 0.74	15.60 ± 2.24	15.94 ± 1.56	15.68 ± 0.57
WBC [10^9/l]	CON	9.88 ± 2.11	7.76 ± 0.87	5.78 ± 1.02	6.46 ± 1.97	8.96 ± 2.50	8.08 ± 1.28
	PRO	10.12 ± 0.94	8.34 ± 1.05	8.70 ± 1.46	7.06 ± 1.92	9.70 ± 0.91	10.04 ± 2.53
LYM [10^9/l]	CON	5.00 ± 1.03	5.40 ± 1.31	3.48 ± 0.90	3.11 ± 0.81	4.17 ± 0.87	4.10 ± 0.83
	PRO	4.99 ± 0.27	3.70 ± 0.68	4.10 ± 0.60	4.14 ± 1.01	4.83 ± 0.91	4.81 ± 1.01
BAND [10^9/l]	CON	0.11 ± 0.03	0.11 ± 0.03	0.09 ± 0.04	0.12 ± 0.03	0.18 ± 0.16	0.10 ± 0.02
	PRO	0.30 ± 0.15	0.26 ± 0.18	0.25 ± 0.22	0.26 ± 0.22	0.11 ± 0.04	0.12 ± 0.11
NEUT [10^9/l]	CON	3.93 ± 1.01	2.13 ± 0.68	2.07 ± 0.61	2.64 ± 1.58	3.98 ± 1.94	2.98 ± 1.17
	PRO	4.03 ± 0.57	4.14 ± 0.94	4.26 ± 1.66	2.68 ± 1.33	4.52 ± 0.94	4.49 ± 1.61
EOS [10^9/l]	CON	0.82 ± 0.56	0.60 ± 0.68	0.23 ± 0.15	0.62 ± 0.67	0.66 ± 0.75	0.85 ± 0.58
	PRO	0.72 ± 0.65	0.69 ± 0.43	0.55 ± 0.56	0.25 ± 0.27	0.31 ± 0.24	0.53 ± 0.23

Abbreviations: BDO, Before Dry-Off; d-14, 14-10 days prepartum; d+7, 5-10 days postpartum; w+4, 3-4 weeks postpartum; w+8, 7-8 weeks postpartum; w+12, 11-12 weeks postpartum. WBC, White Blood Cells; RBC, Red Blood Cells; HGB, Hemoglobin; HCT, Hematocrit; MCV, Mean Corpuscular Volume; MCH, Mean Corpuscular Hemoglobin; MCHC, Mean Corpuscular Hemoglobin Concentration; PLT, Platelets; BAND, Band Neutrophils; NEUT, Segmented Neutrophils; EOS, Eosinophils; LYM, Lymphocytes. PRO, Experimental group; CON, Control group.

Supplementary Table 5. Serum protein metabolism parameters (mean ± SD) in control (CON; *n* = 5) and probiotic-supplemented (PRO; *n* = 5) cows at different time points relative to calving. Standardized effect sizes are reported in Supplementary Table 9.

	**Group**	**BDO**	**d-14**	**d+7**	**w+4**	**w+8**	**w+12**
TP [g/l]	CON	83.66 ± 8.11	65.84 ± 7.27	68.94 ± 3.03	73.98 ± 1.02	77.12 ± 3.17	79.96 ± 9.26
	PRO	82.80 ± 6.03	68.08 ± 1.71	75.20 ± 7.31	83.86 ± 4.87	83.78 ± 7.01	83.54 ± 8.28
ALB [g/l]	CON	43.06 ± 1.38	38.66 ± 0.66	39.50 ± 0.87	41.18 ± 1.82	42.66 ± 3.05	42.30 ± 3.12
	PRO	44.02 ± 0.48	42.28 ± 1.39	42.18 ± 4.21	43.72 ± 1.81	44.32 ± 2.11	44.30 ± 0.70
GLOB [g/l]	CON	40.60 ± 7.72	27.18 ± 6.86	29.44 ± 2.23	32.80 ± 1.69	34.46 ± 4.70	37.66 ± 10.55
	PRO	38.78 ± 5.88	25.92 ± 1.71	33.02 ± 5.16	40.20 ± 4.89	38.86 ± 7.62	39.44 ± 7.65
A/G Ratio	CON	1.09 ± 0.19	1.48 ± 0.28	1.35 ± 0.07	1.26 ± 0.12	1.26 ± 0.23	1.19 ± 0.33
	PRO	1.16 ± 0.18	1.64 ± 0.15	1.30 ± 0.20	1.10 ± 0.15	1.18 ± 0.23	1.16 ± 0.22
UREA [mg/dL]	CON	35.56 ± 12.50	35.70 ± 7.60	38.58 ± 13.12	34.62 ± 14.25	27.28 ± 4.59	27.28 ± 4.59
	PRO	32.36 ± 0.91	37.34 ± 9.79	28.10 ± 9.56	25.40 ± 4.22	28.00 ± 5.24	29.80 ± 1.92

Abbreviations: BDO, Before Dry-Off; d-14, 14-10 days prepartum; d+7, 5-10 days postpartum; w+4, 3-4 weeks postpartum; w+8, 7-8 weeks postpartum; w+12, 11-12 weeks postpartum. TP, Total Protein; ALB, Albumin; GLOB, Globulin; A/G Ratio, Albumin to Globulin Ratio; UREA, Urea, PRO, Experimental group; CON, control group.

Supplementary Table 6. Serum free amino acid concentrations (mean ± SD), raw *p* values, and Benjamini–Hochberg false-discovery-rate-adjusted *q* values in control (CON; *n* = 9) and probiotic-supplemented (PRO; *n* = 10) cows at 7–8 weeks postpartum. Standardized effect sizes for all individual amino acids and summary variables are reported in Supplementary Table 10.

Category	Metabolites	Control	Experimental	p value	q value
Essential AA	His	37.56 ± 19.94	25.70 ± 19.89	0.236	0.527
	Ile	96.33 ± 38.63	91.10 ± 17.25	0.715	0.822
	Leu	59.67 ± 25.45	79.30 ± 34.69	0.462	0.664
	Lys	61.11 ± 18.35	74.30 ± 20.93	0.414	0.664
	Met	12.33 ± 5.45	15.50 ± 4.81	0.302	0.579
	Phe	41.11 ± 7.85	43.70 ± 6.88	0.458	0.664
	Thr	88.44 ± 21.24	90.50 ± 18.00	0.824	0.861
	Trp	16.67 ± 13.71	26.60 ± 12.30	0.045*	0.218
	Val	169.78 ± 54.53	169.40 ± 37.78	0.775	0.849
Non-Essential AA	Ala	156.67 ± 30.29	179.70 ± 33.97	0.137	0.394
	Arg	157.11 ± 30.58	144.50 ± 41.07	0.456	0.664
	Asn	20.11 ± 5.01	13.30 ± 3.53	0.004**	0.092
	Asp	6.56 ± 5.75	24.80 ± 16.73	0.008**	0.092
	Cys	16.28 ± 20.41	12.30 ± 15.88	0.625	0.757
	Gln	363.44 ± 77.38	280.90 ± 56.16	0.019*	0.146
	Glu	102.33 ± 42.67	68.30 ± 24.64	0.057	0.218
	Gly	429.67 ± 156.71	329.50 ± 68.84	0.105	0.345
	Ser	69.22 ± 32.16	74.60 ± 20.87	1.000	1.000
	Tyr	38.11 ± 15.57	46.30 ± 7.67	0.180	0.460
Other metabolites	Cit	55.44 ± 20.38	50.60 ± 7.24	0.515	0.697
	Etn	43.33 ± 64.60	16.40 ± 10.43	0.252	0.527
	Orn	24.33 ± 12.42	37.00 ± 14.61	0.057	0.218
	Tau	44.22 ± 12.85	45.40 ± 14.16	0.623	0.757
Summary	Sum EAA	740.11 ± 140.41	760.60 ± 150.84	0.903	—
	Sum NEAA	1210.44 ± 236.36	1054.40 ± 155.99	0.116	—
	TAA	2181.22 ± 348.18	1999.90 ± 247.76	0.216	—

Abbreviations: Ala, alanine; Arg, arginine; Asn, asparagine; Asp, aspartic acid; Cit, citrulline; Cys, cysteine; EAA, essential amino acids; Etn, ethanolamine; Gln, glutamine; Glu, glutamic acid; Gly, glycine; His, histidine; Ile, isoleucine; Leu, leucine; Lys, lysine; Met, methionine; NEAA, nonessential amino acids; Orn, ornithine; Phe, phenylalanine; Ser, serine; TAA, total amino acids; Tau, taurine; Thr, threonine; Trp, tryptophan; Tyr, tyrosine; Val, valine. Raw *p* values are shown for the original between-group comparisons. Benjamini–Hochberg-adjusted *q* values were calculated across the 23 individual amino acids; summary variables were not included in the correction family. Asterisks refer to the corresponding raw *p* values.

Supplementary Table 7. Milk production and composition parameters (mean ± SD) during the first 105 days in milk (DIM) in probiotic-supplemented (PRO; *n* = 5) and control (CON; *n* = 5) cows. Standardized effect sizes are reported in Supplementary Table 11.

Parameter evaluated	Experimental	Control
Cumulative milk yield over 105 DIM (kg/cow)	3278 ± 386.6	3721 ± 366.4
Mean daily milk yield (kg/cow/day)	31.21 ± 3.69	35.43 ± 3.49
Cumulative fat yield per cow (kg/cow)	118.46 ± 13.37	131.51 ± 8.28
Mean milk fat content (%)	3.58 ± 0.2	3.45 ± 0.27
Cumulative protein yield per cow (kg/cow)	102.6 ± 11.06*	121.2 ± 9.38
Mean milk protein content (%)	3.15 ± 0.12	3.16 ± 0.07
Cumulative milk dry matter yield per cow (kg/cow)	398.6 ± 44.86	463.48 ± 35.02
Mean milk dry matter content (%)	12.25 ± 0.34	12.09 ± 0.35

Values are presented as mean ± standard deviation. The asterisk indicates a p value < 0.05.

Supplementary Table 8. Analytic cohorts, sampling schedules, and outcome domains.

Outcome domain	Analytic set	Sampling schedule	Interpretive role
Randomized enrollment	CON, n = 10; PRO, n = 10	Allocation at enrollment	Source cohort for all prespecified analytic sets
Free serum amino acids	CON, n = 9; PRO, n = 10	One sample at 7–8 weeks postpartum	Primary metabolic domain; one CON cow was culled for an unrelated reason
Hematology and serum protein indices	CON, n = 5; PRO, n = 5	Before dry-off; 10–14 days prepartum; 5–10 days, 3–4 weeks, 7–8 weeks, and 11–12 weeks postpartum	Nested longitudinal subcohort
Milk production, composition, and SCC	CON, n = 5; PRO, n = 5	Monthly AT4 test days during the first 105 DIM	Same nested longitudinal subcohort

The longitudinal subcohort was nested within the full randomized cohort and did not constitute an independent animal set. Abbreviations: CON, control group; DIM, days in milk; PRO, probiotic-supplemented group; SCC, somatic cell count.

Supplementary Table 9. Standardized between-group effect sizes for serum protein indices (PRO, n = 5; CON, n = 5).

Parameter	Time point	Cohen's d	Hedges' g (95% CI)
TP [g/l]	BDO	-0.12	-0.11 (-1.23, 1.02)
TP [g/l]	d-14	0.42	0.38 (-0.76, 1.51)
TP [g/l]	d+7	1.12	1.01 (-0.24, 2.21)
TP [g/l]	w+4	2.81	2.54 (0.85, 4.16)
TP [g/l]	w+8	1.22	1.11 (-0.16, 2.32)
TP [g/l]	w+12	0.41	0.37 (-0.78, 1.49)
ALB [g/l]	BDO	0.93	0.84 (-0.37, 2.01)
ALB [g/l]	d-14	3.33	3.01 (1.14, 4.80)
ALB [g/l]	d+7	0.88	0.80 (-0.41, 1.96)
ALB [g/l]	w+4	1.40	1.26 (-0.04, 2.51)
ALB [g/l]	w+8	0.63	0.57 (-0.60, 1.71)
ALB [g/l]	w+12	0.88	0.80 (-0.41, 1.96)
GLOB [g/l]	BDO	-0.27	-0.24 (-1.36, 0.89)
GLOB [g/l]	d-14	-0.25	-0.23 (-1.35, 0.90)
GLOB [g/l]	d+7	0.90	0.81 (-0.39, 1.98)
GLOB [g/l]	w+4	2.02	1.83 (0.37, 3.22)
GLOB [g/l]	w+8	0.70	0.63 (-0.55, 1.77)
GLOB [g/l]	w+12	0.19	0.17 (-0.95, 1.29)
A/G Ratio	BDO	0.38	0.34 (-0.80, 1.46)
A/G Ratio	d-14	0.71	0.64 (-0.54, 1.79)
A/G Ratio	d+7	-0.33	-0.30 (-1.42, 0.84)
A/G Ratio	w+4	-1.18	-1.06 (-2.27, 0.19)
A/G Ratio	w+8	-0.35	-0.31 (-1.43, 0.83)
A/G Ratio	w+12	-0.11	-0.10 (-1.21, 1.03)
UREA [mg/dL]	BDO	-0.36	-0.33 (-1.45, 0.81)
UREA [mg/dL]	d-14	0.19	0.17 (-0.96, 1.29)
UREA [mg/dL]	d+7	-0.91	-0.82 (-1.99, 0.39)
UREA [mg/dL]	w+4	-0.88	-0.79 (-1.95, 0.41)
UREA [mg/dL]	w+8	0.15	0.13 (-0.99, 1.25)
UREA [mg/dL]	w+12	0.72	0.65 (-0.53, 1.79)

Note: Effect sizes were calculated from the displayed group means and standard deviations. Positive values indicate higher values in PRO; negative values indicate lower values in PRO.

Supplementary Table 10. Standardized between-group effect sizes for serum free amino acids and summary variables (PRO, n = 10; CON, n = 9).

Category	Metabolite	Cohen's d	Hedges' g (95% CI)
Individual amino acid	His	-0.60	-0.57 (-1.44, 0.32)
Individual amino acid	Ile	-0.18	-0.17 (-1.03, 0.69)
Individual amino acid	Leu	0.64	0.61 (-0.28, 1.49)
Individual amino acid	Lys	0.67	0.64 (-0.26, 1.52)
Individual amino acid	Met	0.62	0.59 (-0.30, 1.47)
Individual amino acid	Phe	0.35	0.34 (-0.54, 1.20)
Individual amino acid	Thr	0.11	0.10 (-0.76, 0.96)
Individual amino acid	Trp	0.76	0.73 (-0.17, 1.61)
Individual amino acid	Val	-0.01	-0.01 (-0.87, 0.85)
Individual amino acid	Ala	0.71	0.68 (-0.22, 1.56)
Individual amino acid	Arg	-0.35	-0.33 (-1.19, 0.54)
Individual amino acid	Asn	-1.59	-1.52 (-2.50, -0.50)
Individual amino acid	Asp	1.43	1.36 (0.37, 2.32)
Individual amino acid	Cys	-0.22	-0.21 (-1.07, 0.66)
Individual amino acid	Gln	-1.23	-1.18 (-2.11, -0.22)
Individual amino acid	Glu	-0.99	-0.95 (-1.85, -0.02)
Individual amino acid	Gly	-0.84	-0.81 (-1.70, 0.11)
Individual amino acid	Ser	0.20	0.19 (-0.67, 1.05)
Individual amino acid	Tyr	0.68	0.65 (-0.25, 1.53)
Individual amino acid	Cit	-0.32	-0.31 (-1.17, 0.56)
Individual amino acid	Etn	-0.60	-0.57 (-1.45, 0.32)
Individual amino acid	Orn	0.93	0.89 (-0.03, 1.79)
Individual amino acid	Tau	0.09	0.08 (-0.78, 0.94)
Summary	Sum EAA	0.14	0.13 (-0.73, 0.99)
Summary	Sum NEAA	-0.79	-0.75 (-1.64, 0.15)
Summary	TAA	-0.61	-0.58 (-1.45, 0.31)

Effect sizes were calculated from the displayed group means and standard deviations. Positive values indicate higher values in PRO; negative values indicate lower values in PRO.

Supplementary Table 11. Standardized between-group effect sizes for milk production and composition outcomes (PRO, n = 5; CON, n = 5).

Outcome	Cohen's d	Hedges' g (95% CI)
Cumulative milk yield over 105 DIM (kg/cow)	-1.18	-1.06 (-2.27, 0.20)
Mean daily milk yield (kg/cow/day)	-1.18	-1.06 (-2.27, 0.20)
Cumulative fat yield per cow (kg/cow)	-1.17	-1.06 (-2.26, 0.20)
Mean milk fat content (%)	0.55	0.49 (-0.66, 1.62)
Cumulative protein yield per cow (kg/cow)	-1.81	-1.64 (-2.97, -0.24)
Mean milk protein content (%)	-0.10	-0.09 (-1.21, 1.03)
Cumulative milk dry matter yield per cow (kg/cow)	-1.61	-1.46 (-2.75, -0.10)
Mean milk dry matter content (%)	0.46	0.42 (-0.73, 1.54)

Effect sizes were calculated from the displayed group means and standard deviations. Positive values indicate higher values in PRO; negative values indicate lower values in PRO.
